# Effect of Carbon Ion Radiation Induces Bystander Effect on Metastasis of A549 Cells and Metabonomic Correlation Analysis

**DOI:** 10.3389/fonc.2020.601620

**Published:** 2021-03-02

**Authors:** Zhen Yang, Qiuning Zhang, Hongtao Luo, Lihua Shao, Ruifeng Liu, Yarong Kong, Xueshan Zhao, Yichao Geng, Chengcheng Li, Xiaohu Wang

**Affiliations:** ^1^ The Basic Medical College of Lanzhou University, Lanzhou, China; ^2^ Institute of Modern Physics, Chinese Academy of Sciences, Lanzhou, China; ^3^ Department of Oncology, Lanzhou Heavy Ion Hospital, Lanzhou, China; ^4^ Department of Oncology, The First School of Clinical Medicine, Lanzhou University, Lanzhou, China

**Keywords:** carbon ions, metastasis, radiation-induced bystander effect, liquid phase mass spectrometry, metabonomics

## Abstract

**Objective:**

To analyze the effect of carbon ion (^12^C^6+^) radiation may induce bystander effect on A549 cell metastasis and metabonomics.

**Methods:**

A549 cell was irradiated with carbon ion to establish the clone survival model and the transwell matrix assay was applied to measure the effect of carbon ion on cell viability, migration, and invasion, respectively. Normal human embryonic lung fibroblasts (WI-38) were irradiated with carbon ions of 0 and 2 Gy and then transferred to A549 cell co-culture medium for 24 h. The migration and invasion of A549 cells were detected by the Transwell chamber. The analysis of metabonomic information in transfer medium by liquid phase mass spectrometry (LC-MS), The differential molecules were obtained by principal pomponent analysis (PCA) and the target proteins of significant differences (*p* = 1.7 × 10^−3^) obtained by combining with the STICH database. KEGG pathway was used to analyze the enrichment of the target protein pathway.

**Results:**

Compared with 0 Gy, the colony formation, migration, and invasion of A549 cells were significantly inhibited by carbon ion 2 and 4 Gy irradiation, while the inhibitory effect was not significant after 1 Gy irradiation. Compared with 0 Gy, the culture medium 24 h after carbon ion 2 Gy irradiation significantly inhibited the metastasis of tumor cells (*p* = 0.03). LC-MS analysis showed that 23 differential metabolites were obtained in the cell culture medium 24 h after carbon ion 0 and 2 Gy irradiation (9 up-regulated and 14 down-regulated). Among them, two were up-regulated and two down-regulated (*p* = 2.9 × 10^−3^). 41 target proteins were corresponding to these four differential molecules. Through the analysis of the KEGG signal pathway, it was found that these target molecules were mainly enriched in purine metabolism, tyrosine metabolism, cysteine and methionine metabolism, peroxisome, and carbon metabolism. Neuroactive ligand-receptor interaction, calcium signaling pathway, arachidonic acid metabolism, and Fc epsilon RI signaling pathway.

**Conclusion:**

The bystander effect induced by 2 Gy carbon ion radiation inhibits the metastasis of tumor cells, which indicates that carbon ions may change the metabolites of irradiated cells, so that it may indirectly affect the metabolism of tumor cell growth microenvironment, thus inhibiting the metastasis of malignant tumor cells.

## Introduction

In 2018, there were 18.1 million new cases of cancer worldwide, of which the incidence of lung cancer was 11.6% and the mortality rate was 18.4% (1). However, the metastasis of lung cancer is a major cause of treatment failure (2). Carbon ions with high LET rays (^12^C^6+^) can inhibit the metastasis of tumor cells by their advantages in physics and biology (3, 4). Radiation induces bystander effect (RIBE) refers to the plethora of biological phenomena occurring in non-irradiated cells as a result of signal transmission from an irradiated cell (5). The study of proton targeting cancer cells and unirradiated normal fibroblasts also found an induced bi-directional bystander effect, and RIBE was detected *in vitro*, 3D tissues and mouse models (6).Growing evidence show that bystander responses can be regulated by four mechanisms-(i) gap junction intercellular communication (ii) communication of soluble factors released by irradiated cells or organs (iii) clastogenic factors and exosomes (7, 8).

Recently, it has been found that reactive oxygen species (ROS) (9), superoxide dismutase (SOD), nitric oxide (NO) (10), cyclooxygenase 2 (COX2), CD95 ligand (Fals), transforming growth factor-β1 (TGFβ1), and tumor necrosis factor-α(TNF-α) (11) play an important roles in RIBE. Among all these factors, some are soluble factors (TGFβ1 and TNF-α), some are exosome released factors (Fals), others are common factors of both (ROS, SOD, NO, and COX2), are very similar effect to RIBE. The maximum molecular weight allowed to pass through the pores of gap junctional intercellular communication (GJIC) is 1,000 to 1,500 Da, which allows ions, small molecular metabolites and second messengers to communicate directly between cells. The molecular weight of soluble factors is between 1,000 and 10,000 kDa. Molecules smaller than 1,000 Da are selected as the main metabolic factors in this study, so they belong to clastogenic factors and exosomes-mediated bystander effect factors (12). At the same time, the GJIC also plays an interesting role in RIBE (13). After GJIC inhibition, the fractionated doses of proton radiation-induced less DNA damage than single-dose radiation in the secondary bystander effects (14). The mechanism of the radiation-induced bystander effect, whether involving cell-cell contact or mediated by soluble factors, is not clear, is likely to be complex, and involves multiple pathways (15). At present, there are two main research reports—(i) For cells in direct contact, bystander signaling can occur through GJIC. Gap junctions are multimeric protein channels between cells that allow transmission of signaling molecules. Key ions and metabolites that are known to be transmitted through GJIC include ions such as Ca^2+^, nucleotides, peptides and other secondary messengers (16). (ii) Its mechanism that radiation exposure can result in the release of soluble (clastogenic) factors or exosomes into the microenvironment that are capable of inducing chromosome damage in cultured cells, thus changing the biological characteristics of unirradiated cells. Among them, the studies on cytokine signal transduction and production of reactive oxygen species and nitrogen species are relatively clear (17).

Although there are many reports about RIBE research, only a few studies about the relationship of carbon ion bystander effects (CIBE) and malignant tumor cell metastasis. It has been found that glutamate involved in tumorigenesis, and glutamate concentration plays a key role in the invasion and migration of pancreatic cancer cells (18). Understanding radiation-induced signaling pathways is essential for developing new strategies in both cancer radiotherapy and the prevention of radiation carcinogenesis (19). Therefore, in our study, we used metabonomics techniques were used to analyze the metabolic molecules of carbon ion-induced fine radiation bystander effects, meanwhile we combined with bioinformatics methods to screen differential metabolites and possibly target molecules to explain the effect and potential effects of carbon ion radiation bystander effects on non-small cell lung cancer (NSCLC) cell metastasis.

## Materials and Methods

### Cell Culture

Human lung adenocarcinoma cell line A549 and normal human embryonic fibroblasts WI-38 cells were purchased from the American Type Culture Collection (ATCC). The cells were maintained in RPMI1640 medium supplemented with 10% (v/v) fetal bovine serum (FBS), 100 U/ml penicillin and 100 mg/ml streptomycin (Life Technologies) in a humidified atmosphere of 5% (v/v) CO_2_ and 95% (v/v) air at 37°C. Every other day, the medium was changed, and cells in the logarithmic growth phase (1 × 10^7^ cells were harvested at 70% confluence) were used in the experiments.

### Irradiation Condition

Heavy ions were obtained from the carbon ion (^12^C^6+^) beam of the Deep Therapy Terminal, Institute of Modern Physics, Chinese Academy of Sciences (HIRFL-CSR) (Ray parameters: energy of 100 MeV, the dose rate of 1 Gy/min, broadened Bragg peak of 5 mm, radiation field of 5 cm × 5 cm), for cell irradiation. Irradiation doses were 0, 1, 2, and 4 Gy. The experiment was repeated four times.

### Medium Transfer Protocol

Exponentially growing cells were irradiated with carbon ion, and then incubated in fresh medium to allow for the release of soluble bystander factors. After carbon ion treatment (counted as 0 h), irradiation conditioned medium was harvested at the time points of 24 h, respectively. These irradiation conditioned medium were filtered through 0.22-μm filter (Millpore, USA) to remove any floating cells and cellular debris. These irradiation conditioned medium were used to culture the nonirradiated cells (bystander cells). For the controls in all experiments, the irradiation conditioned medium were collected from a parallel culture that had not been irradiated.

### Colony Formation Assay

Cells in logarithmic growth were detached with 0.25% trypsin and then triturated into single cells and centrifuged. The number of cells was counted with a counting board. A549 cells were inoculated separately in a six-well plate after being resuspended in culture medium containing 10% FBS: 0 Gy group: 400 cells/well, 1 Gy group: 800 cells/well, 2 Gy group: 1,000 cells/well, and 4 Gy group, 4,000 cells/well. Three replicate wells were used for each group. After overnight inoculation, the cells were exposed to 0, 1, 2, and 4 Gy carbon ion rays and cultured in a cell incubator for 13 days. When the cell colonies were visible to the naked eye, approximately 50 cells were counted under the microscope, and the experiment was terminated. Next, the cells were fixed with 4% polyformaldehyde (500 μl) for 15 min, stained with a crystal violet solution (500 μl) for 15 min, and observed. Plating efficiency (PE) = colony number/inoculation number × 100%, and survival fraction (SF) = colony rate in the experimental group/colony rate in the control group × 100%. The cell dose survival curve was generated using the formula SF=e^^^(−(αD + βD^^^2)) and GraphPad Prism 6 software. The experiment was repeated three times.

### Cell Counting kit-8 (CCK-8) Assay

A549 cells grown in wells were detached with trypsin and resuspended in a small amount of culture medium, and the cells were then counted. Next, 100 μl (approximately 10,000 cells) of cell suspension was added to each well of a 96-well plate. Six replicate wells were used for each group, and the culture plate was placed in an incubator for pre-culture for 24 h (37°C, 5% CO_2_) to allow the cells to adhere. The cells were exposed to carbon ion radiation when the cells reached 50% to 60% confluence. Cell proliferation was detected at 24, 48, and 72 h after irradiation. Each well was incubated with 10 μl CCK-8 solution for 2 h. The optical density (OD) value of each well was detected by a microplate reader (450 nm). The experiment was repeated three times.

### Detection of A549 Metastasis by Direct and Indirect Effects of Carbon Ion Irradiation

The A549 cells were irradiated with different doses of carbon ions, and then Transwell was used to detect migration and invasion. Migration and invasion of A549 were detected by transwell chamber after carbon ion irradiation. The Transwell assay for the ability of invasion was performed as the following protocol: the polycarbonate membranes with an 8-μm pore (Corning, USA) were placed on 24-well Transwell plates (Corning, USA), The lower side of the filter was precoated with 10μg/mL collagen type I-C (Millpore, USA). After incubation for 3 h, the number of cells that had migrated to the lower side was counted in five independent fields. These experiments were repeated a minimum of four times. The Transwell assay for the ability of migration was performed as the following protocol: Millpore filters with 8-lm pores (Millpore, USA) were precoated with 100 ml of 0.1 μg/ml Matrigel (BD, USA). After incubation for 24 h, the number of cells that had invaded to the lower side through the pores was counted in five independent fields. In both assays, FBS was used as a chemoattractant. These experiments were repeated a minimum of four times. The irradiated cells were prepared by trypsin digestion and cells (3 × 10^4^) were resuspended in medium RPMI 1640 containing 1% (v/v) FBS and added to the top chamber. A culture medium containing 5% (v/v) FBS was then added to the bottom chamber. Meanwhile, for determining the influence of conditioned medium on the metastasis of bystander cells, medium of unirradiated cells in Transwells chamber was replaced with irradiation conditioned mediumn collected nonirradiated cells. For the controls in experiments, the irradiation conditioned mediumn were collected from a parallel culture that had not been irradiated. Other experimental conditions are the same as those for the detection of radiation-induced direct effects. Cell motility/migration was measured as the number of cells migrated from a defined area of the uncoated microfilter through micropores in the given time, 24 h. The cells that migrated to the lower surface of the membrane were fixed with methanol for 10 min and stained with 0.5% (w/v) crystal violet (BD Biosciences) for 30 min.

### Metabonomics Analysis of RIBE Factors

After WI-38 cells were irradiated by carbon ion 2 Gy, the culture medium was collected at 24 h, and the differential factors in the culture medium were detected by LC-MS.LC-TOFMS (Agilent,1290 Infinity LC, 6530 UHD, and Accurate-Mass Q-TOF/MS) was used for the metabonomic profiling of all samples in the study. The profiling procedure (sample preparation, metabolite separation and detection, metabonomic data preprocessing, metabolite annotation, and statistical analysis for biomarker identification) was performed following our previously published protocols with minor modifications. The separation column was a C18 column (Agilent, 100 mm × 2.1 mm, 1.8 μm). The chromatographic separation conditions are as follows: column temperature 40 °C, flow rate 0.4 ml/min; mobile phase A: water + 0.1% formic acid, B: acetonitrile + 0.1% formic acid; according to a certain gradient elution procedure. The injection volume is 4ul and the temperature of the automatic injector is 4°C.

### Data Analysis and Statistics

Mean values of control and treated samples were compared using one-way analysis of variance (ANOVA) where P < 0.05 was considered to be statistically significant. All annotated metabolites from GC-TOFMS data sets were combined and exported to SIMCA-P+ 13.0 (Umetrics AB, Umea, Sweden) and R 3.6.1 for multivariate statistical analysis. Partial least squares discriminant analysis (OPLS-DA) and orthogonal partial least squares-discriminant analysis (OPLS-DA) was performed to discriminate between carbon 2 and 0 Gy. Initially, an exploratory multivariate analysis was applied to log2-transformed ratios scaled to unit variance. The multiple test problem was addressed by calculating the false discovery rate (FDR) using the Benjamini and Hochberg method.

## Results

### Carbon Ion Beams Inhibited Cell Colony Formation and Cell Proliferation

**Figure 1:**
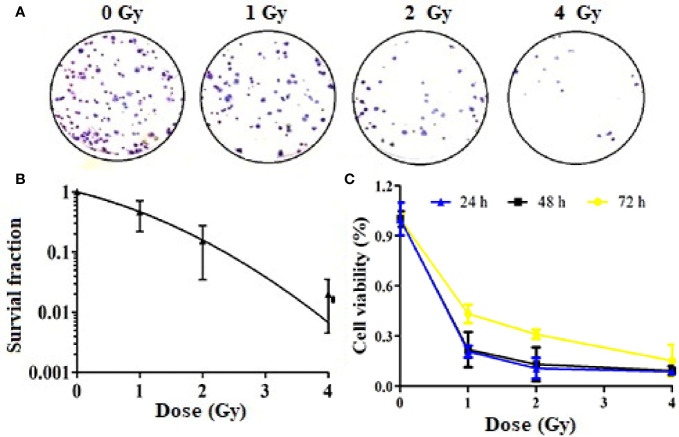
Inhibition of Colony formation and proliferation of A549 cells by carbon ions [**(A)** colony formation, **(B)** survival fraction, **(C)** inhibition of cell viability].

We also evaluated the effect of carbon ion beams on the colony formation ability of A549 cells. The results confirmed that the colony formation ability was significantly lower in cells treated with 1, 2, and 4 Gy than in cells treated with 0 Gy (**Figure 1A**). There was a positive correlation between the clone inhibition rate and the carbon ion irradiation dose. The survival of A549 cell lines decreased after 1 Gy irradiation, but there was no significant difference (**Figure 1B**). However, after 2 and 4 Gy irradiation, cell survival decreased significantly (*p* = 0.02). The plating effiency of each group is 24.5, 8.75, 1.90, and 2.04%. The CCK-8 assay results revealed different growth changes after 24, 48, and 72 h. After 1 Gy irradiation, A549 cell proliferation was significantly inhibited at 24 h, and this inhibition increased in a dose-dependent manner. With the extension of time after irradiation, the ability of cell proliferation was gradually restored (**Figure 1C**).

### Effect of Carbon Ion on Metastasis of A549 Cells

**Figure 2:**
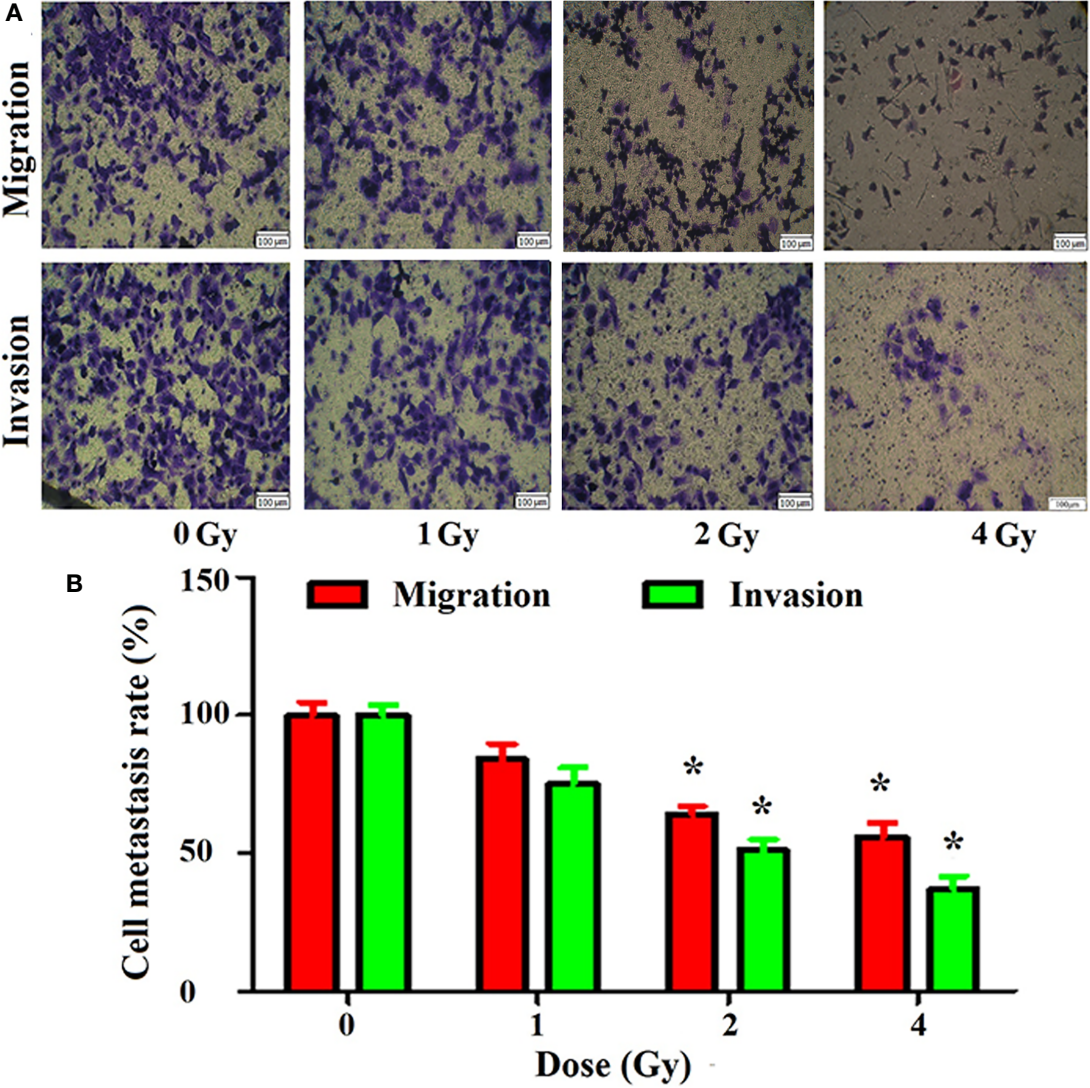
Inhibition of migration and invasion of A549 cells by carbon ions [**(A)** migration and invasive staining, **(B)** metastasis difference analysis] *p < 0.05.

The results of the cell Transwell chamber assay revealed that carbon ion rays inhibited cell migration and invasion compared with the control group (**Figure 2A**). Carbon ions at 2 and 4 Gy significantly inhibited the migration and invasion of A549 cells (p = 0.01) (**Figure 2B**). The migration and invasion abilities of A549 cells were decreased in 1 Gy group with respect to the 0 Gy group, but it was not significantly different (p = 0.07).

### Migration and Invasion of A549 Cells After Culture Medium Transfer

**Figure 3:**
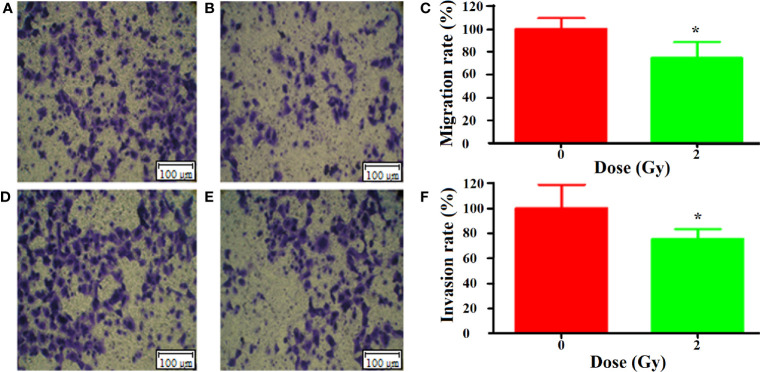
Bystander effect of carbon ion (^12^C^6+^) 2 Gy irradiation on metastasis of A549 cells [**(A)** 0 Gy migration, **(B)** 2 Gy migration, **(C)** mobility difference analysis; **(D)** 0 Gy invasion, **(E)** 2 Gy invasion, **(F)** analysis of the difference of invasion rate]. "*p < 0.05.

According to the direct effect results obtained above, we chose 24 h after carbon ion 2 Gy irradiation as the dose and time for the study of bystander effects. The results of Transwell showed that the migration of A549 cells was significantly inhibited by the culture medium irradiated by carbon ion 2 Gy compared with that of 0 Gy (**Figures 3A, B**), and the migration of A549 cells was significantly inhibited by the culture medium irradiated by carbon ion 2 Gy (*p* = 0.03) (**Figure 3C**). Compared with 0 Gy (**Figures 3D, E**), the culture medium irradiated by 2 Gy carbon ions for 24 h could significantly inhibit the invasion of A549 cells (*p* = 0.05) (**Figure 3F**).

### Liquid Chromatography Coupled to Mass Spectrometry (LC-MS) Detection and Principal Component Analysis (PCA)

**Figure 4:**
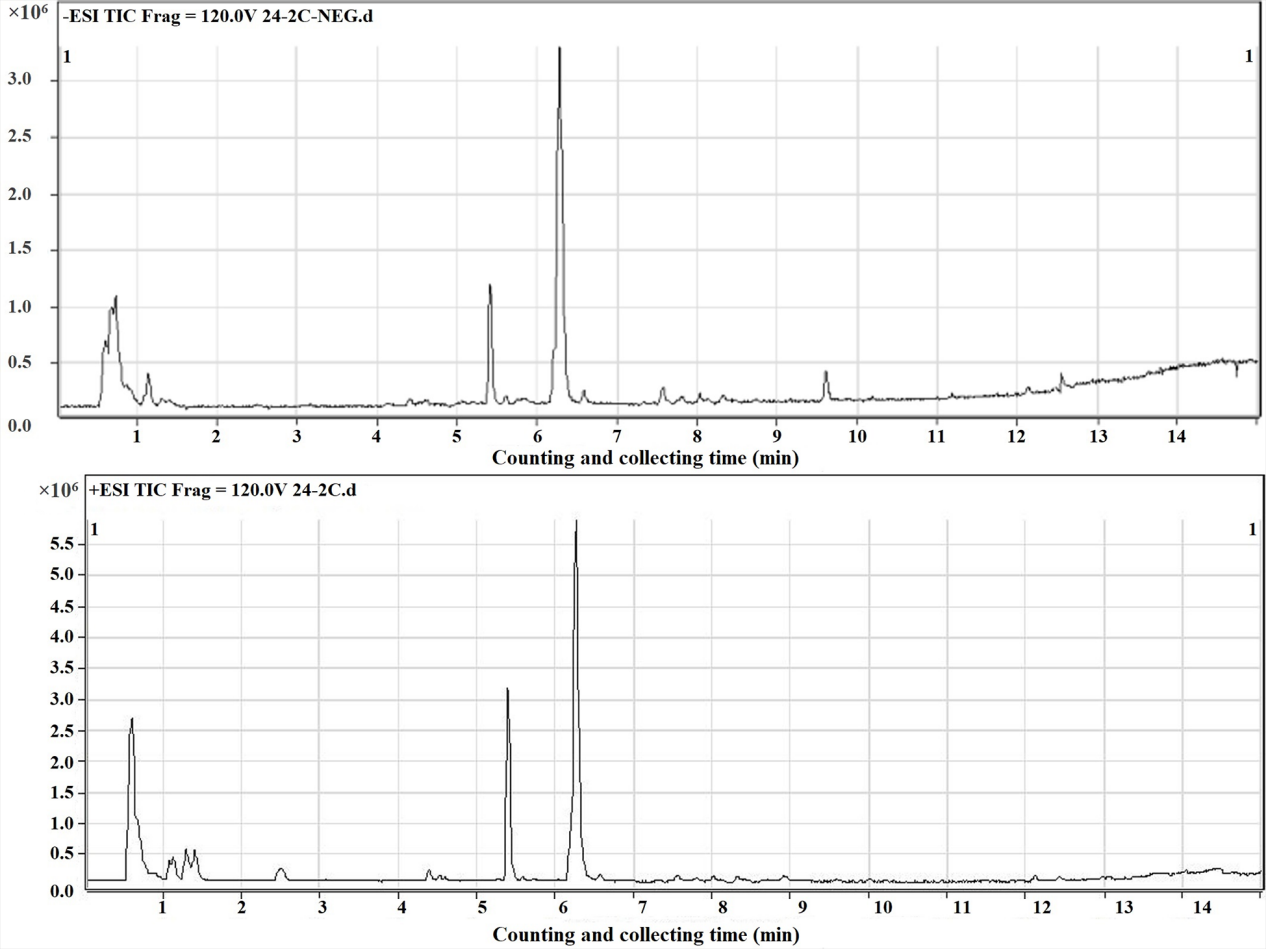
Total ion current chromatogram of carbon ion 2 Gy irradiated sample.

**Figure 5:**
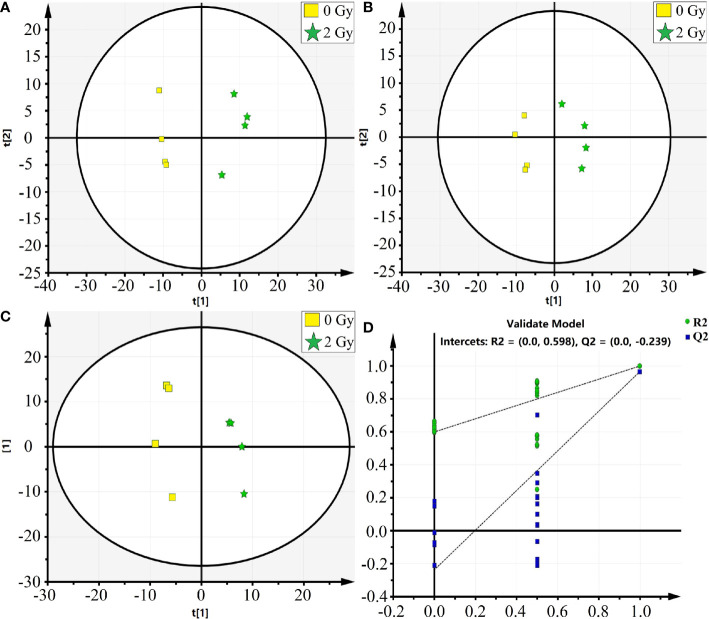
The PCA and OPLS-DA analysis on the differential metabolic molecules after carbon Ion irradiation [**(A)** PCA analysis, **(B)** PAS-DA analysis, **(C)** OPLS-DA analysis, **(D)** PLS-DA score plot, in which the abscissa represents similarity with the original model, the ordinate represents R2Y and Q2 values].

In this study, the mass spectrometer uses both positive and negative ion modes for quality detection, and the response value in the positive ion mode is generally low, and the fragment ion information is lacking, so the negative ion mode is finally selected for analysis. Total ion chromatography of 0 and 2 Gy was obtained by ultra-high-performance liquid chromatography coupled to quadruple time-of flight mass spectrometer (UPLC/Q-TOF/MS) analysis (**Figure 4**). The reproducibility and stability of the instrument were preliminarily examined. A total of 361 metabolites were structurally identified from the LC-MS data based on spectral matching against a reference database. Of the 361 metabolites, 162 metabolites were distributed in 0 Gy group and 176 in 2 Gy group. The molecular characterization is completed through the online database (http://metlin.scripps.edu/) (comparing the exact molecular weight mass of mass spectrometry).To analyze the different molecules between the two groups, the data obtained were unsupervised by PCA and PLS-DA. The scoring graph of the first principal component (*t*[*1*]) direction and the second principal component (*t*[2]) direction are shown in Fig. 5A. The results showed that the metabolic components of the two groups were separated well, indicating that different doses of carbon ion radiation could significantly change the metabolites.

### Analysis and Screening of Metabolites

In **Figure 5A** we show Principal Component Analysis (PCA) of the carbon ion irradiation along with the control group. The negative mode of PLS-DA shows that the current PLS-DA model (R2X = 0.813, R2Y = 0.984, Q2 = 0.965) is very reliable (**Figure 5B**), which is suitable for explaining the metabolic differences between the two groups and finding the differences in expressed metabolites between the two groups. The supervised OPLS-DA method was further used to model and analyze the differential metabolites (**Figure 5C**). The results showed that one principal component and one orthogonal component were obtained in negative mode (R2X = 0.887, R2Y = 0.965, Q2 = 0.863). Significantly altered irradiation metabolites with the variable importance in the projection (VIP) threshold (VIP > 1) in the above-mentioned OPLS-DA model, as well as the Mann-Whitney U test (p < 0.05), were selected differentially expressed molecular induced by radiation. The cross-validation Q2 (cum) in the PLS-DA model was 0.965 (**Figure 5D**). Heatmap analysis of these differential proteins corresponding to volcano plot. It could be observed that there were 162 and 176 important differentially expressed metabolites in the 0 Gy group and 2 Gy group, respectively. Among these differential metabolites those differential co-expression metabolites in top 23 VIP value were collected (**Table 1**).

**Table 1:** Differential carbon ion irradiaton induced metabolites between 2 and 0 Gy controls in the Learning Group.

Name	Mass	VIP	T-test	Fold change*
Pipecolic acid	129.079	1.433	0.000	10.341
Methylglutaric acid	146.058	1.212	0.001	1.548
Leukotriene C4	625.293	1.007	0.000	3.512
Ribose	150.053	1.057	0.002	1.105
Betaine	117.107	1.360	0.000	−0.791
PS(21:0)	567.348	1.259	0.009	0.634
Niacin	123.032	1.279	0.007	0.507
Homophenylalanine	179.095	1.035	0.002	0.286
Serotonine	176.095	1.365	0.012	0.129
Aminoadipic acid	161.069	1.627	0.040	−0.190
Indoleacrylic acid	187.064	1.307	0.042	−0.201
Isoleucine/Leucine	131.095	1.205	0.018	−0.216
Benzoic acid	122.037	1.477	0.019	−0.279
Valine/Norvaline	117.079	1.341	0.003	−0.343
Ketodeoxycholic acid	390.278	1.138	0.003	−0.369
Pyroglutamic acid	129.043	1.318	0.003	−0.450
Talopyranose	180.201	1.240	0.001	−1.57
Sphinganine	301.299	1.443	0.001	−0.529
PC(16:0)	495.333	1.512	0.004	−0.746
Creatinine	113.059	1.512	0.000	−0.794
Xanthine	152.033	1.453	0.010	−0.639
Glycocholic acid	465.318	1.160	0.002	0.004
Phenylacetic acid	136.128	1.114	0.012	−0.348

*The fold change (FC) was calculated by the average value of 2 Gy group to that of control group. FC with a value larger than 0.0 indicates a significantly higher level of the carbon irradiation induced metabolite in 2 Gy while a FC value lower than 0.0 indicates a lower level compared to 0 Gy controls. Student t test was used for statistical comparisons.

### Target and Network Prediction of Differential Molecules and Analysis of KEGG Pathway

**Figure 6:**
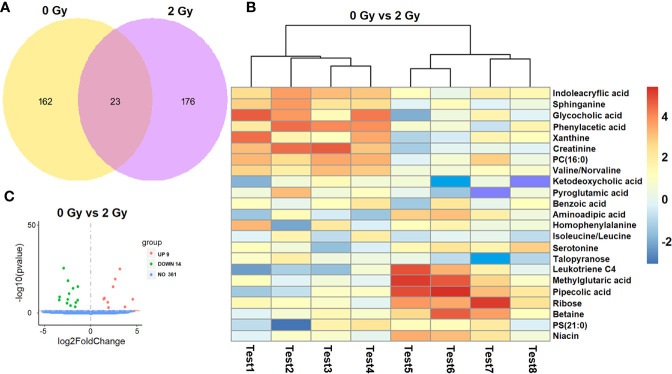
Screening analysis of a differential molecules after different doses of carbon ion irradiation [**(A)** Venn diagram of co-expression molecule, **(B)** expression changes of differential molecules in two groups. **(C)** screening of differential molecules].

**Figure 7:**
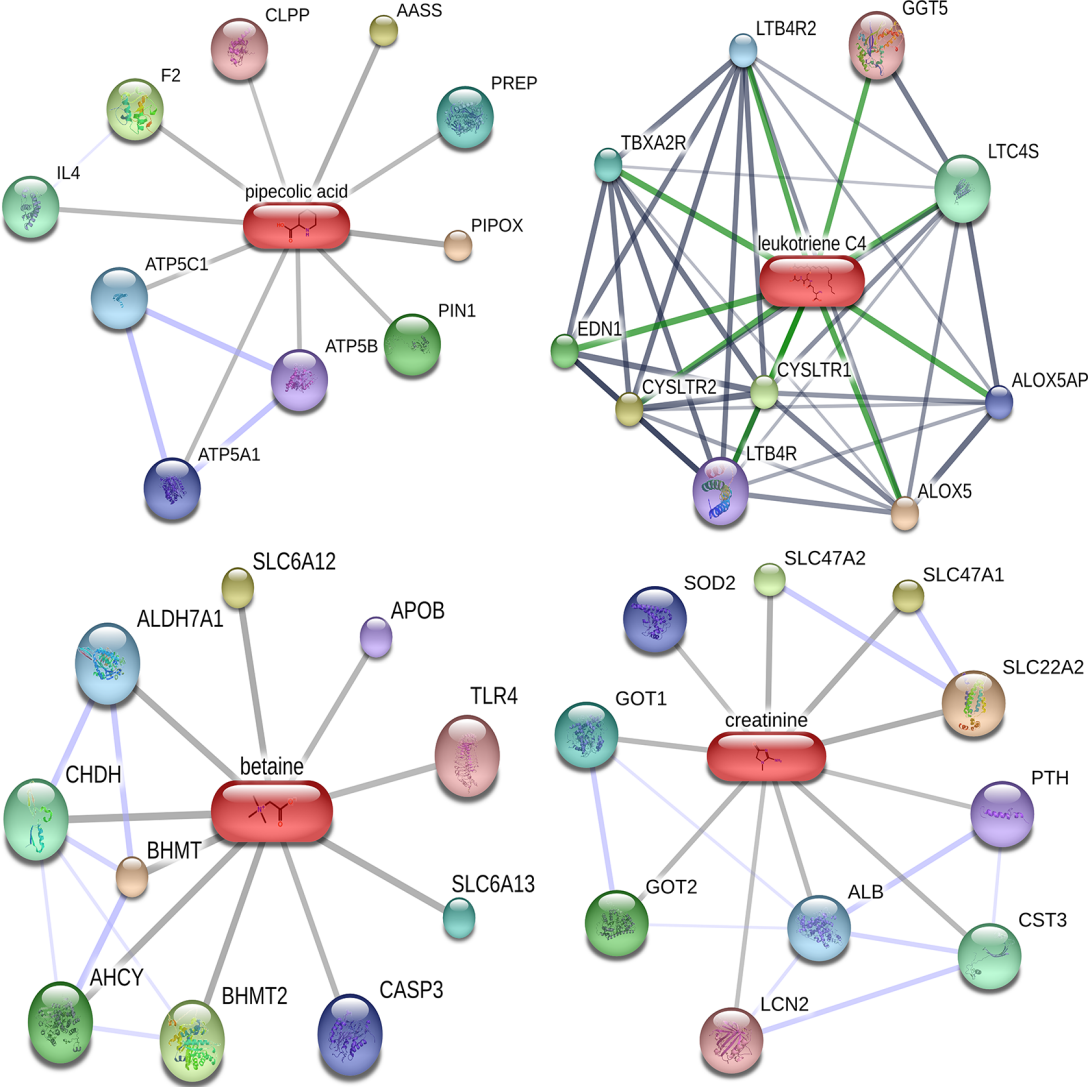
Target molecular network diagram for predicting four significantly different metabolites in Cytoscape.

**Figure 8:**
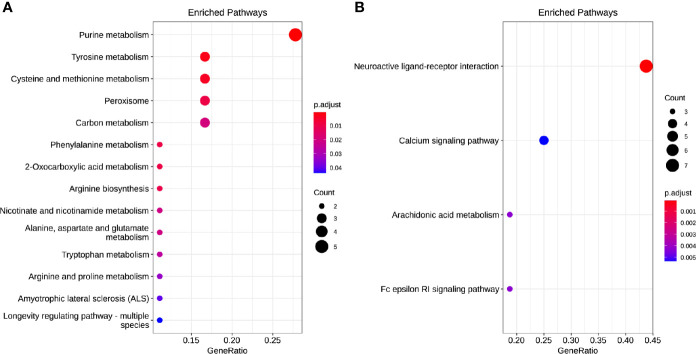
The enrichment model of target molecules in the KEGG pathway [**(A)** downregulated and **(B)** up-regulated].

Of the 384 metabolites, 185 metabolites were normally distributed in 0 Gy group and 199 in 2 Gy group. Combined with fold change value, the expression changes of differential molecules were analyzed (**Figures 6A, B**). The results of co-expression analysis shows that 23 differential molecules were identified, nine molecules were up-regulated, and 14 were down-regulated (**Table 1**). Further analysis showed that the expression of 14 metabolites such as Betain, Creatinine, Xanthine, Sphinganine, Talopyranose, Ketodeoxycholic acid and Pyroglutamic acid decreased after 2 Gy irradiation (**Figure 6C**). The expression of nine metabolic molecules such as Pipecolic acid, Leukotriene C4, Ribose, Niacin, Homophenylalanine, and Serotonine was up-regulated (**Figure 6B**). A total of 41 potential target proteins of four significant differences molecules (Betain and Creatinine, Pipecolic acid, and Leukotriene C4) were obtained from the STITCH database (**Figure 7**). Through KEGG pathway enrichment analysis, we discovered the top 20 significantly metabolism regulated KEGG pathways (**Figure 8**). it was found that 20 target proteins of the two down-regulated differential molecules, Betain and Creatinine were mainly enriched in the Purine metabolism, Tyrosine metabolism, Cysteine, and methionine metabolism, Peroxisome, and Carbon metabolism pathway (**Figure 8A**). The 21 target proteins of two up-regulated differential molecules of Pipecolic acid and Leukotriene C4 were mainly enriched in Neuroactive ligand-receptor interaction, Calcium signaling pathway, Arachidonic acid metabolismand Fc epsilon RI signaling pathway (**Figure 8B**).

## Discussion

The main aim of this study was to assess the global metabonomics and metastasis changes in A549 cell line after carbon ion radiation. Ionizing radiation is defined as a radiation which has sufficient energy to ionize biological molecules. Carbon ion therapy is more advantageous than conventional radiotherapy because of the protection of normal tissues adjacent to the tumor during dose-escalation therapy (20). In this study, we first investigated the direct effects of carbon ions on A549 cells, and carbon ion radiation caused changes in the colony formation and proliferation of A549 cells. We observed that the colony formation rate of A549 cells irradiated with carbon ion rays was significantly lower, and the colony formation rate and survival fraction of A549 cells were significantly lower after irradiation at each dose (1, 2, and 4 Gy) compared with 0 Gy, especially this change was most pronounced at 4 Gy. Carbon ions could inhibit the proliferation of A549 cells, and the activity of A549 cells was the lowest at 24 h after irradiation, as we previously observed in esophageal cancer (21). Secondly, we studied the effect of carbon ion irradiation on the metastasis of A549 cells, and analyzed the migration and invasion of A549 cells after carbon ion irradiation. We observed that carbon ion 2 and 4 Gy significantly inhibited the migration and invasion of A549 cells 24 h after irradiation compared with 0 Gy. Different LET rays can inhibit the migration and invasion of tumor cells (22), with a dependent relationship between the inhibitory ability and irradiation dose (23). At the same time, the indirect effect of carbon ion irradiation on A549 cells was studied. The culture medium collected 24 h after 2 Gy carbon ion irradiation on human normal cells (WI-38) was transferred to A549 cells by the method reported by (13, 24), and the metastasis changes of A549 cells were observed again. The co-culture method was used to study the bystander effect induced by iron ion irradiation in AG01522 cells and its relationship with time (25). It was found that the medium of A549 cells after carbon ion radiation was used as a medium, thus changing the biology of non-irradiated cells. Carbon ions induce upsurge in bystander cell death in lung carcinoma cells, but manifest Type II bystander effects in hepatoma cells (26). It is possible that the primary bystander cells themselves are capable of producing secondary bystander signals to their neighboring cells and creating the radiation-induced Type II bystander effect.

In this study, we found that carbon ion radiation changed the metabolite secretion of irradiated cells, thus affecting the biological behavior of unirradiated cells. Generally, X-rays promote migration and invasiveness under normoxic conditions, and carbon ions can significantly reduce migration (27) and invasion of tumor cells *in vitro* and vivo (23, 28). Further, we used metabonomic analysis to confirm that the culture medium collected from carbon ion irradiated WI-38 cells is related to the decrease of metastatic potential of A549 cells. Compared with untreated cells, the biological process of culture medium related to tumor metastasis after carbon ion irradiation. It had shown that fractionated doses of protons caused less DNA damage in the secondary bystander WI-38 cells compared to a single radiation dose, where the means differ by 20%. This may also be due to the involvement of primary bystander cells releasing secreted diffusible factors into shared growth media (14).Our studies have shown that carbon ions of 2 Gy can induce up-regulation of metabolites in irradiated cells, such as pipemidic acid, leukotriene C4, ribose, niacin, homophenylalanine, and 5-hydroxytryptamine. Exosomes are closely related to lipids, lipid transporters and lipid metabolic enzymes. Radiation-induced up-regulation of cytokine and death ligand secretion by glioblastoma cells established the conditions for radiation-induced bystander response of NSC that was mediated mostly soluble factors released in the media by cancer cells (29). Cholesterol, phospholipids and sphingomyelin are the key substances for the formation of exocrine phospholipid bilayers. Exocrine induces the biosynthesis of leukotriene (LTs). Leukotriene is an effective pro-inflammatory lipid intermediary. Neutral sphingomyelinase overexpressed in exosomes can catalyze the production of ceramide and lead to neuronal apoptosis (30). The results of this study showed that the medium of WI-38 cells 24 h after irradiation with 2 Gy of carbon ions significantly inhibited the migration and invasion of A549 cells, which may be that carbon ion radiation bystander changed the metabolic pattern of A549 cells, thereby inhibiting tumor metastasis. We hypothesize that metabolites induced after carbon ion radiation alter the microenvironment of non-irradiated cells (energy changes, small molecule expression, etc.), which affects the biological behavior of tumor cells. Previous studies implicated in irradiated WI-38 and irradiated A549 cells demonstrated metabolic interference between irradiated and non-irradiated cells (31).

Metabolic conversion is one of the markers of cancer (32). Therefore, our study clearly showed that carbon ions can change the metabolic factors of tumor, and these metabolic factors regulate the characteristics of tumor by changing the metabolic mode of tumor to act on targeted molecules. Many reports have demonstrated the involvement of piperidic acid and its derivatives can be used as inhibitors of matrix metalloproteinases (33) and related transferases for the synthesis of anti-tumor drugs. In order to maintain the dynamic balance of the microenvironment, these cancer cells secrete more lactic acid, thus reducing the pH value in the tumor microenvironment and enhancing the metastatic potential of these cancer cells (34). Furthermore, we demonstrated that CIRE induced changes in glycolysis, cyanoamino acid, and citric acid metabolic pathways. Studies have found that the Warburg effect not only gives the energy needed for the growth of cancer cells, but also promotes the process of metastasis (35). The Warburg effect is a metabolic phenomenon in cancer cells, where even in the presence of adequate oxygen, there is high glycolytic activity (aerobic glycolysis) and lactate production. Our results show that CIBE can significantly inhibit the metastasis of A549 cells, so it can be speculated that carbon ion radiation can reduce the energy metabolism of cancer cells. We speculated that Betaine may inhibit tumor glucose metabolism and play an anti-tumor effect. At the same time, Betaine has significant difference in the metabolism of liver cancer, so it can be used as an excellent and potential diagnostic index (36). Our analysis founds that the differential metabolites induced by carbon ion radiation interact with other molecules (IL4, TLR4, SOD2, SLC22A2, etc.) to regulate the metastasis of A549 cells. Carbon ions not only directly act on tumor cells and change the phenotype of tumor cells, but also affect the phenotype of non-irradiated cells near irradiated cells. This may be attributed to the activation of multiple signal transduction pathways in bystander cells. It has been shown that indicate that heavy ions inactivate clonogenic potential of bystander cells, and that the time course of the response to heavy ions differs between irradiated and bystander cells (37).

These metabolisms allow the tumor microenvironment to be under acidic conditions that can inhibit the migration and invasion of tumor cells by dependent on the p53 and Caspase 3 pathways for necrosis and apoptosis (38). We have found that down-regulated molecule (betaine and creatinine) is mainly involved in Purine metabolism, Tyrosine metabolism, Cysteine and methionine metabolism, Peroxisome and Carbon metabolism pathway. Of these molecules we know, arachidonic acid has immunomodulatory and anticancer effects (39). Glucose reprogramming and glutamine metabolism not only provide the factors needed for tumor cell growth, but also reduce the pressure on the tumor microenvironment through redox reactions (40). The inhibition degree of these irreversible catabolic enzymes is highly related to tumor invasiveness, and can independently predict clinical outcomes (41). Up-regulation of molecular (Pipecolic acid and Leukotriene C4) is mainly involved in Neuroactive ligand-receptor interaction, Calcium signaling pathway, Arachidonic acid metabolism and Fc epsilon RI signaling pathway. Researcher have previously shown that RIBE responses differ among patients. Among which, it is found that Pyruvate, Lactic acid, Arginine and Glutamic acid decreased significantly, Tryptophan metabolism up-regulated, Histamine and Glutamate metabolic pathway, Tricarboxylic acid cycle and intestinal flora metabolic were disorders in colorectal cancer (42). Furthermore, there is an evidence that Serine, Glycine and one-carbon metabolism are involved in the synthesis of proteins, nucleotides and phospholipids, and related enzymes regulate the proliferation and metastasis of hepatocellular carcinoma (43). As we mentioned above, multiple signaling pathways involving metabolic molecules that cause CIBE are associated with patient inflammation, immune response and lung cancer risk prediction as well as other diseases. Calcium signaling pathway and inflammasome are closely related to immune response (44). The observations that immune prognostic biomarkers in the microenvironment are involved in Neuroactive ligand-receptor interaction pathway in osteosarcoma (45), and that pathway and gated ion channel activity are associated with the risk of lung cancer (46). Given these results, when taken together, the CIBE signals transit either through ICM milieu, may be dependent on cell type and radiation quality.

## Conclusion

Through this study, we prove the potential value of metabonomics after carbon ion radiation. The bystander effects induced by carbon ion radiation may change the non-irradiated tumor microenvironment, thus increasing or decreasing the corresponding metabolites. These metabolites can act on targeted molecules and finally show the ability of collective complex signal regulatory network to act on the metastasis and invasion of malignant tumor cells. The further study of radiation bystander effect and malignant tumor metastasis and its mechanism is expected to provide a scientific basis for the optimization of clinical tumor radiotherapy and radiation protection. A better understanding of the cellular and molecular mechanisms of the bystander phenomenon, together with evidence of their occurrence *in vivo*, will allow us to formulate a more accurate model in assessing the health effects of low doses of high LET radiation.

## Data Availability Statement

The raw data supporting the conclusions of this article will be made available by the authors, without undue reservation.

## Author Contributions

Conception and design: ZY, XW, and QZ. Administrative support: XW, HL, and QZ. Experiment operation: HL, ZY, LS, YK, RL, XZ, YG, and CL. Data analysis and interpretation: HL, QZ, ZY, and LS. Manuscript writing: all authors. Final approval of manuscript: all authors. All authors contributed to the article and approved the submitted version.

## Funding

The article were supported by the Lanzhou Innovation and Entrepreneurship Talent Project (award number: 2017-RC-23/2020-RC-113) and the Science and Technology Plan Project of Chengguan District, Lanzhou (award number: no. 2020-2-2-5).

## Conflict of Interest

The authors declare that the research was conducted in the absence of any commercial or financial relationships that could be construed as a potential conflict of interest.
