# Corrigendum: Effect of Carbon Ion Radiation Induce Bystander Effect on Metastasis of A549 Cells and Metabonomic Correlation Analysis

**DOI:** 10.3389/fonc.2021.783377

**Published:** 2021-10-12

**Authors:** Zhen Yang, Qiuning Zhang, Hongtao Luo, Lihua Shao, Ruifeng Liu, Yarong Kong, Xueshan Zhao, Yichao Geng, Chengcheng Li, Xiaohu Wang

**Affiliations:** ^1^ The Basic Medical College of Lanzhou University, Lanzhou, China; ^2^ Institute of Modern Physics, Chinese Academy of Sciences, Lanzhou, China; ^3^ Lanzhou Heavy Ion Hospital, Lanzhou, China; ^4^ The First Clinical Medical College of Lanzhou University, Lanzhou, China

**Keywords:** carbon Ions, metastasis, radiation-induced bystander effect, LC-MS, metabonomics

In the original article, there was a mistake in **Figure 2A** (Migration1, 2, 4Gy, and Invasion 1,4Gy) and as published. **There was an error during editing and exporting of single picture leading to wrong** **Figure 2** **provided**. The corrected **Figure 2** appears below.

**Figure 2:**
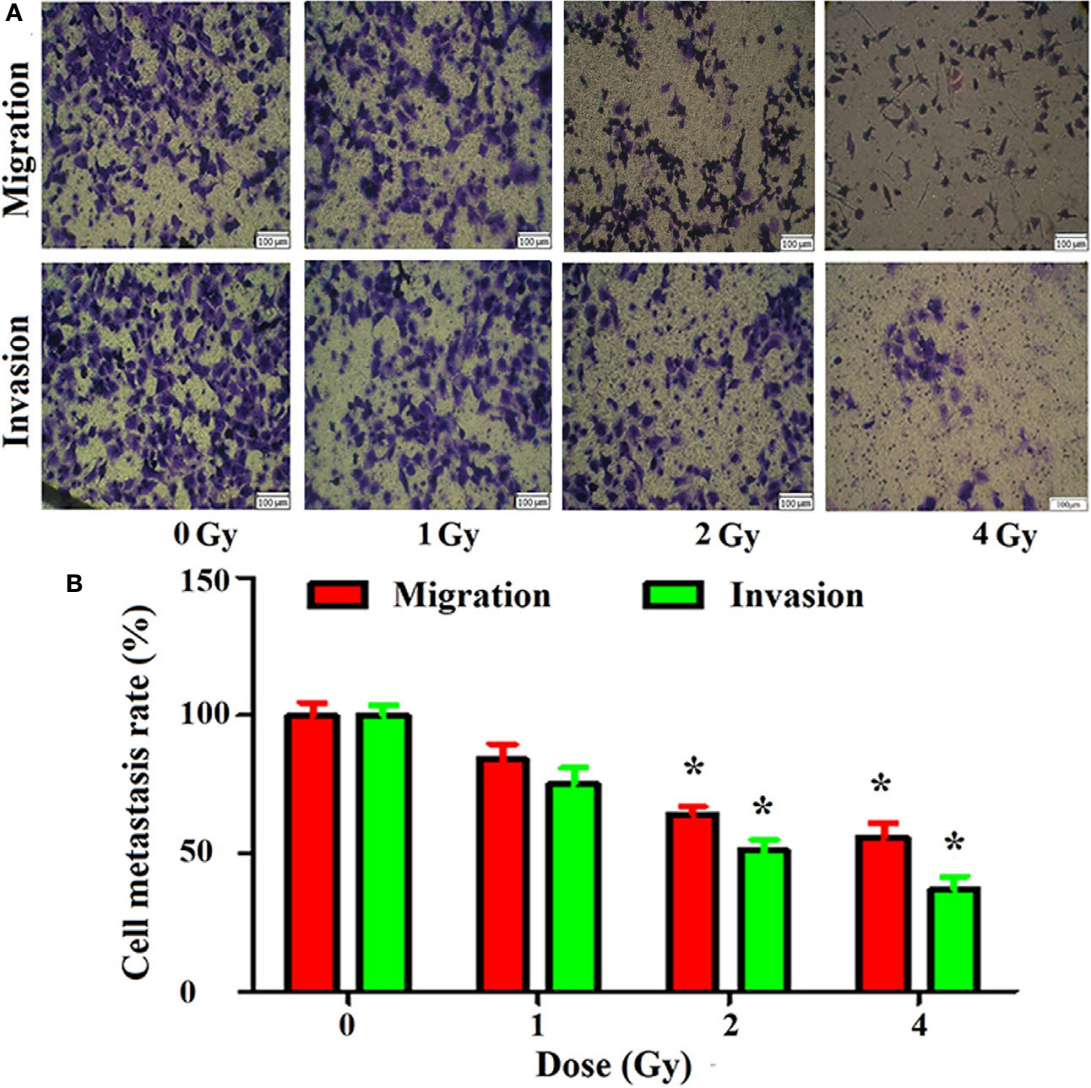
Inhibition of migration and invasion of A549 cells by carbon ions [**(A)** migration and invasive staining, **(B)** metastasis difference analysis] *p < 0.05.

The authors apologize for this error and state that this does not change the scientific conclusions of the article in any way. The original article has been updated.

## Publisher’s Note

All claims expressed in this article are solely those of the authors and do not necessarily represent those of their affiliated organizations, or those of the publisher, the editors and the reviewers. Any product that may be evaluated in this article, or claim that may be made by its manufacturer, is not guaranteed or endorsed by the publisher.

